# Energy down converting organic fluorophore functionalized mesoporous silica hybrids for monolith-coated light emitting diodes

**DOI:** 10.3762/bjoc.13.76

**Published:** 2017-04-25

**Authors:** Markus Börgardts, Thomas J J Müller

**Affiliations:** 1 Institut für Organische Chemie und Makromolekulare Chemie, Heinrich-Heine-Universität Düsseldorf, Universitätsstr. 1, D-40225 Düsseldorf, Germany

**Keywords:** benzofurazane, LED, luminophores, mesoporous silica hybrids, monoliths, Nile red, perylene, postsynthetic grafting, white light emission

## Abstract

The covalent attachment of organic fluorophores in mesoporous silica matrices for usage as energy down converting phosphors without employing inorganic transition or rare earth metals is reported in this article. Triethoxysilylpropyl-substituted derivatives of the blue emitting perylene, green emitting benzofurazane, and red emitting Nile red were synthesized and applied in the synthesis of mesoporous hybrid materials by postsynthetic grafting to commercially available MCM-41. These individually dye-functionalized hybrid materials are mixed in variable ratios to furnish a powder capable of emitting white light with CIE chromaticity coordinates of x = 0.33, y = 0.33 and an external quantum yield of 4.6% upon irradiation at 410 nm. Furthermore, as a proof of concept two different device setups of commercially available UV light emitting diodes, are coated with silica monoliths containing the three triethoxysilylpropyl-substituted fluorophore derivatives. These coatings are able to convert the emitted UV light into light with correlated color temperatures of very cold white (41100 K, 10700 K) as well as a greenish white emission with correlated color temperatures of about 5500 K.

## Introduction

The development of new efficient illumination materials has received increasing attention in the past years [1–3]. For the generation of white light the combination of a UV emitting light source and energy down converting phosphor emitting in the full visible spectrum is an intriguing approach [4–5]. This concept bears the advantage of producing white light with a high color rendering index (CRI) as well as the generation of white light with a warm correlated color temperature (CCT) [4–6]. Usually several UV excitable red, green and blue (RGB) emitting phosphors generally relying on inorganic luminophores, such as rare earth metals as Ce^3+^ or Eu^2+^, are employed for white light generation [3–4,7]. However, the development of purely organic down converting phosphors would offer a sustainable and potentially cost-effective access alternative, eventually starting from renewable organic materials [8–9].

Other than inorganic luminophores these organic analogues cannot simply be intercalated in a robust inorganic host structure and, therefore, ligating materials are required. Since the host structure should not undergo photodegradation, stable structures like silica-based materials, in particular mesoporous silica, offer favorable properties [4,10]. Eventually, organic–inorganic hybrid materials can be easily accessed by covalent ligation of the functional guest chromophore to a silica host matrix [11–12]. As a consequence the inherent robustness and stability of the rigid inorganic silica material can be combined with the functionality and tunability of the organic chromophore. In addition, these mesoporous materials possess defined pore structures in which the organic dye can be homogeneously distributed [13]. Thereby aggregation induced self-quenching and singlet oxygen dependent dye degradation can be efficiently prevented [14–15]. By the rigid silica host framework bimolecular photochemical reactions are also suppressed [16]. Additionally, mesoporous materials possess the crucial advantage that they can be synthesized as transparent monoliths in different shapes [17–19]. Here, we communicate our first efforts to design red, green, and blue light-emitting mesoporous materials from organic chromophores, their blending to white light-emitting silica hybrids based upon additive color mixing, and as a proof of principle, the implementation in a white light emitting monolith for down converting commercially available UV light emitting diodes.

## Results and Discussion

A general approach to mesoporous organo–silica hybrid materials takes advantage of the condensation of triethoxysilyl-functionalized functional organic molecules with inorganic silica hosts [11,20]. Therefore, a terminal triethoxysilyl group has to be coupled to luminescent dyes with blue, green, and red emission characteristics for generating hybrid materials with white light emission based upon additive color mixing. A rapid and versatile functionalization can be achieved upon ligating a triethylsiloxy-functionalized azide and a terminal alkynyl-functionalized luminophore by CuAAC (Cu-catalyzed azide–alkyne cycloaddition) [21–23].

**Scheme 1:**
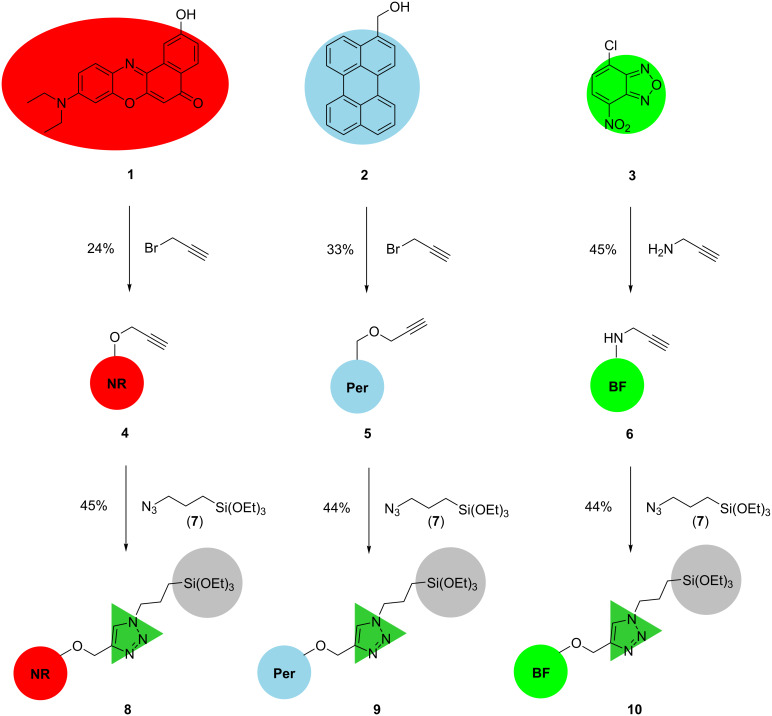
Synthesis of the triethoxysilyl-functionalized dye precursors **8**, **9**, and **10**.

Commencing from a 2-hydroxy-substituted Nile red **1** or 3-hydroxymethylperylene (**2**) the alkyne-substituted fluorophore derivatives **4** and **5** were accessible by Williamson ether synthesis [24]. The alkyne-functionalized green benzofurazane derivate **6** was obtained by nucleophilic aromatic substitution of 4-chloro-7-nitrobenzo[*c*][1,2,5]oxadiazole (**3**) with propargylamine. The alkyne-functionalized derivatives **4**, **5** and **6** were then reacted with azide **7** via CuAAC to furnish the triethoxysilyl-substituted luminophore precursor molecules **8** (red emission), **9** (blue emission), and **10** (green emission) (Scheme 1). In contrast to usually high yields of the CuAAC the precursor synthesis only gives yields between 44 to 45% after purification by column chromatography on silica gel [22–23]. The diminished yields are a consequence of the reaction of the terminal triethoxysilyl groups with the free silanol groups of the silica gel taking place already at room temperature.

**Figure 1:**
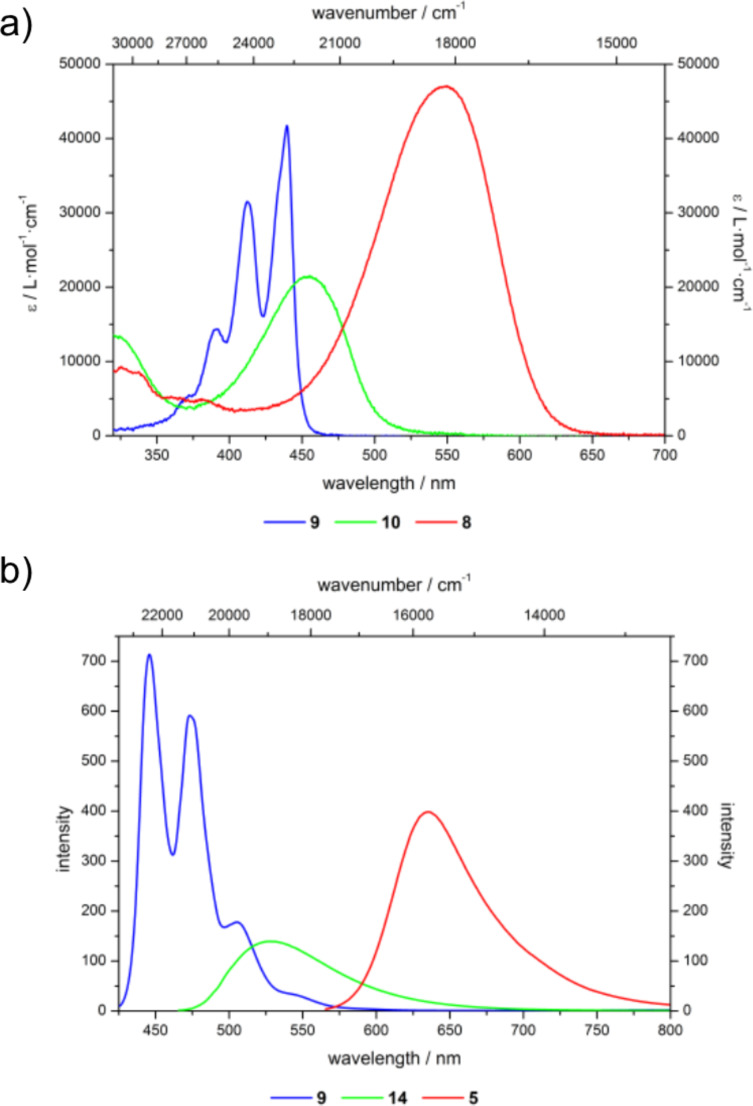
Absorption (a) and emission (b) spectra of perylene **9**, benzofurazane **10**, and Nile red precursors **8** (recorded in ethanol at *T* = 298 K; ([9] = 4.1 × 10^−6^ M, [10] = 4.0 × 10^−6^ M, [8] = 4.1 × 10^−6^ M; λ_exc_(**9**) = 409 nm, λ_exc_(**10**) = 450 nm, λ_exc_(**8**) = 540 nm).

For generating white light emission with good color rendering index and tunable color temperature the characteristics of red emissive Nile red, blue emissive perylene, and green emissive benzofurazane have to be matched with comparable emission intensity. All three chromophores exhibit absorption in the UV–vis region with comparable molar absorption coefficients (Figure 1a). Neglecting energy transfer between the luminophores superposition of the emission spectra of the three dyes covers the whole range of the visible region (400–800 nm) (Figure 1b).

**Figure 2:**
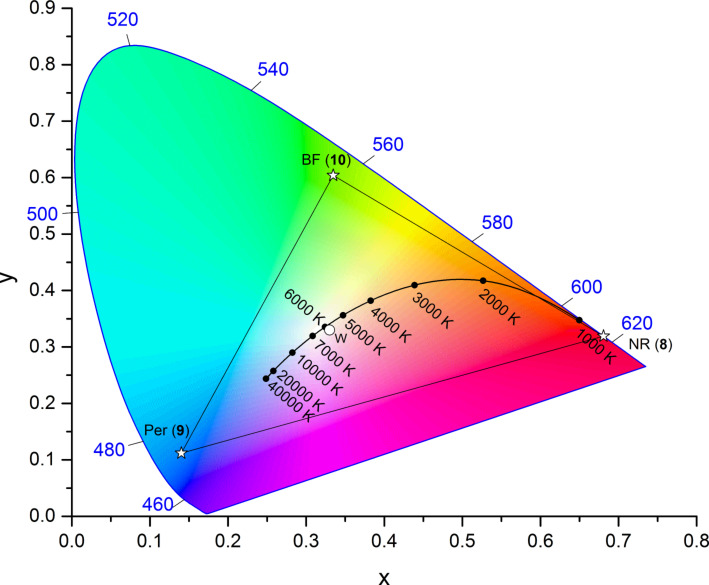
CIE 1931 color space chromaticity diagram (2° observer) with the CIE chromaticity coordinates of the emitting precursors **9** (blue), **10** (green), and **8** (red) in ethanol as well as the color space accessible by mixing these three precursors shown as triangle.

The three dyes were additionally analyzed with respect to their CIE chromaticity coordinates, i.e., their fluorescence color in order to determine the color space which should be accessible by additive color mixing [25–28]. As shown in Figure 2 CIE chromaticity coordinates of the blue, green and red colors were determined as follows: perylene precursor **10**: x = 0.14, y = 0.11; benzofurazane precursor **9**: x = 0.33, y = 0.60; Nile red precursor **8**: x = 0.68, y = 0.32.

Thus, by mixing of the three precursors **8**, **10**, and **9** the color space within the triangle defined by the pure colors should be accessible. As this triangle comprises the spectrum of a black body radiator an emission of white light with tunable color temperature is possible. The synthesis of silica hybrid materials functionalized with a single dye was performed via postsynthetic grafting of commercially available MCM-41 according to literature procedures [12–13,17]. Therefore, the respective dye precursors **9**, **10**, and **8** at variable concentrations were reacted with free silanol groups displayed on the surface of MCM-41 furnishing hybrid materials with different dye loadings in a μmol·g^−1^ range. The amount of dye incorporated into the hybrid material was estimated by UV–vis spectroscopic analysis according to our previous report [24].

The synthesized materials with the corresponding dye loading and solid state quantum yields are summarized in Table 1. Especially for the Nile red-functionalized hybrid materials the highest quantum yield of 23% was obtained at a low dye loading of 1.5 μmol·g^−1^ (**8@MCM-2**). A further increase of Nile red loading causes a drop of quantum yields, presumably due to self-quenching effects.

**Table 1:** Calculated dye loadings and solid state fluorescence quantum yields Φ_f_ of grafted hybrid materials.

**dye@MCM**	calculated dye loading [μmol·g^−1^]	Φ_f_ [%]

**8@MCM**		
**8@MCM-1**	0.6 of **8**	14
**8@MCM-2**	1.5 of **8**	23
**8@MCM-3**	2.9 of **8**	20
**8@MCM-4**	5.9 of **8**	20
**8@MCM-5**	8.8 of **8**	17
**8@MCM-6**	12 of **8**	12
**8@MCM-7**	12 of **8**	11
**8@MCM-8**	18 of **8**	8.6
**8@MCM-9**	23 of **8**	6.7
**9@MCM**		
**9@MCM-1**	0.8 of **9**	5.8
**9@MCM-2**	2.0 of **9**	7.6
**9@MCM-3**	4.0 of **9**	12
**9@MCM-4**	6.0 of **9**	7.3
**9@MCM-5**	8.0 of **9**	6.6
**9@MCM-6**	20 of **9**	7.6
**10@MCM**		
**10@MCM-1**	1.2 of **10**	10
**10@MCM-2**	2.3 of **10**	17
**10@MCM-3**	4.6 of **10**	14
**10@MCM-4**	6.9 of **10**	14
**10@MCM-5**	9.3 of **10**	16
**10@MCM-6**	12 of **10**	19
**10@MCM-7**	16 of **10**	14

**Figure 3:**
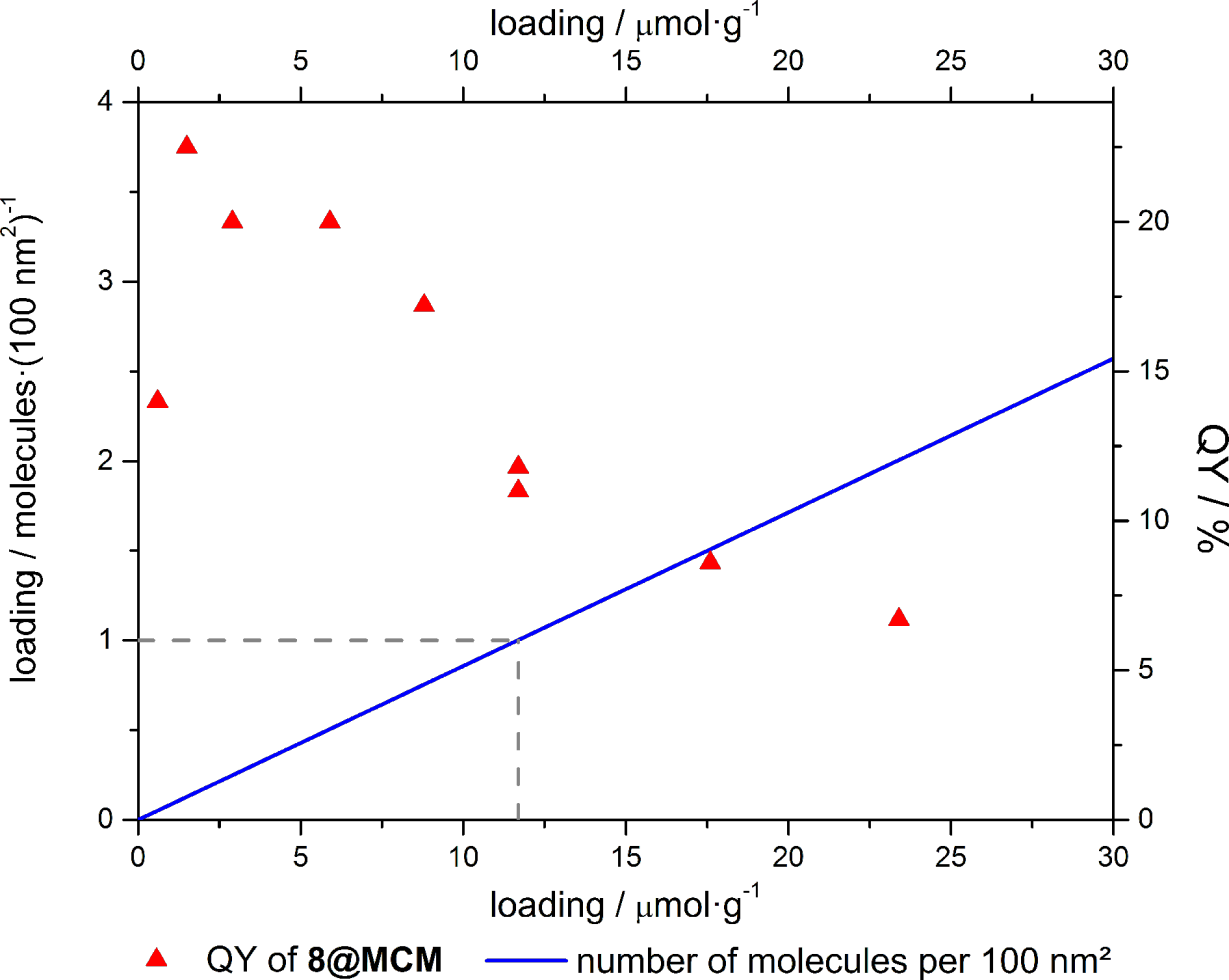
Number of molecules per 100 nm² and quantum yields of **8@MCM** in relation to the loading of hybrid materials.

Based on the assumption that energy transfer typically occurs at distances of less than 10 nm between two dye molecules, self-quenching should be observed if more than one molecule is found in an area of 10 × 10 nm [29]. If dye loadings of the hybrids are correlated with the surface area of the silica material, typically 700 m^2^·g^−1^, self-quenching effects should occur at loadings higher than 10 μmol·g^−1^ as illustrated in Figure 3 [24]. Although aggregation-induced self-quenching can be prevented by covalently ligating dye molecules to the silica matrix, intermolecular energy transfer causing losses in quantum yield can be expected at higher dye loadings.

**Figure 4:**
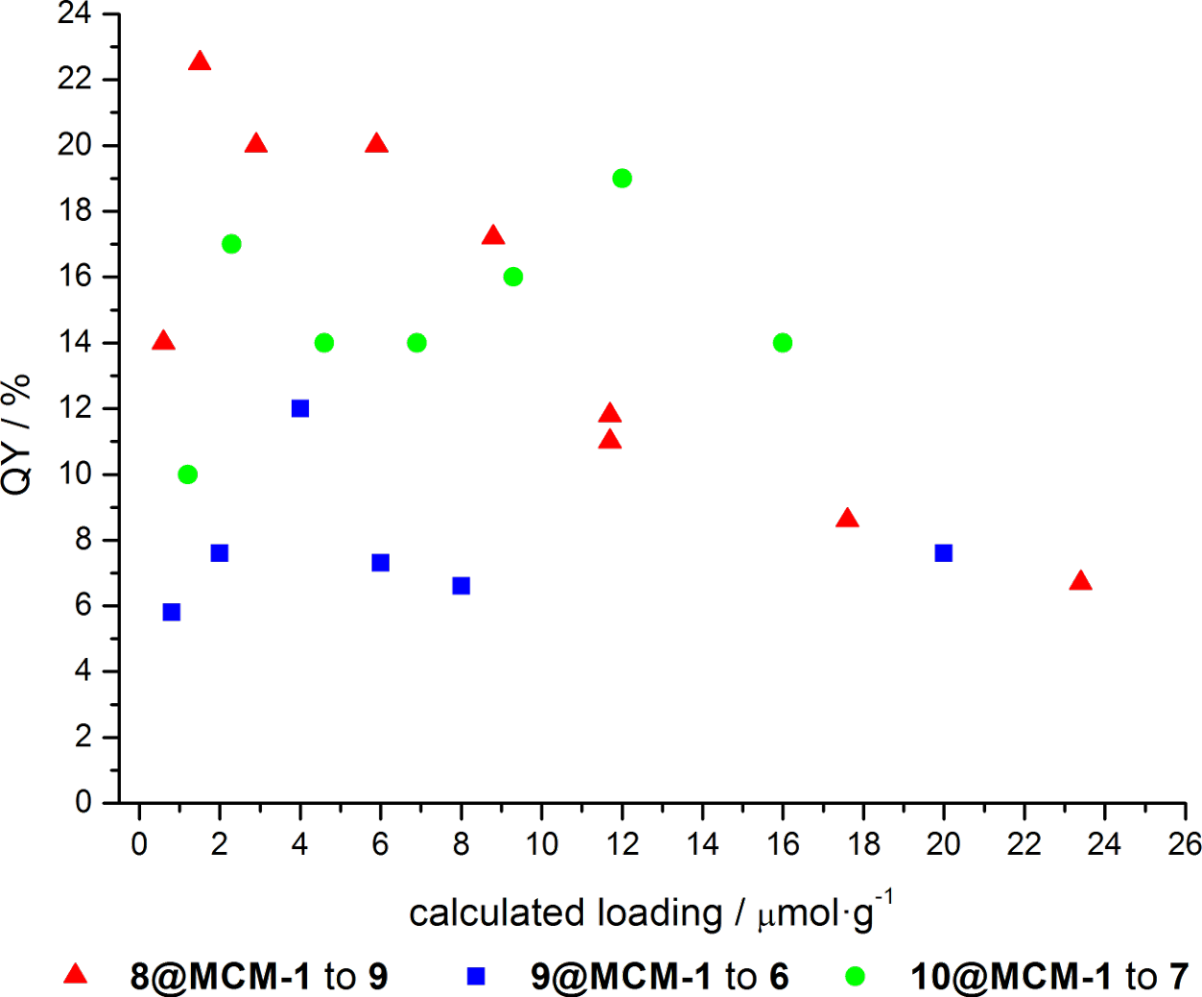
Solid-state fluorescence quantum yields Φ_f_ of grafted hybrid materials in relation to the calculated dye loading (λ_exc_(**9@MCM**) = 409 nm, λ_exc_(**10@MCM**)) = 450 nm, λ_exc_ (**8@MCM**)) = 540 nm).

Likewise, for the perylene and benzofurazane-functionalized hybrid materials **9@MCM-3** and **10@MCM-6** similar dye loadings in the lower μmol·g^−1^ range were ascertained for furnishing optima of their solid-state fluorescence quantum yields Φ_f_ (Figure 4).

The optical properties of the dye-functionalized hybrid materials differ slightly from the properties of their precursor molecules in solution. For all hybrid materials a broad band can be observed in the spectra, which can be attributed to an inhomogeneous line broadening in the solid state. As benzofurazane and Nile red dyes exhibit solvatochomism, their spectral properties are clearly affected by incorporation into the polar silica environment. A detailed study of the spectral properties and solvatochromism of the Nile red hybrid materials in comparison to the precursor molecule **8** was previously reported and extensively discussed [24].

Also for perylene-functionalized hybrids a peculiar influence on the optical properties by incorporation into a silica matrix was found. Most striking is the change of the vibrational fine structure. The solution spectra of the perylene precursor **9** in ethanol show an increasing intensity of the vibrations with increasing wavelength in the absorption spectrum as well as a typical mirror image of the emission spectrum (vide supra Figure 1a and b). But as shown in Figure 7 (vide infra) for the perylene-functionalized hybrid materials, the second vibrational band becomes the most intense in the emission spectrum. This behavior clearly reflects the change in the fluorescence spectra of the free precursor at higher concentrations. This is typical for dyes with small Stokes shifts capable of inner filter and energy transfer effects.

With single dye-functionalized silica hybrid materials in hand displaying highest quantum yields, blends with tunable color temperature should be realized. In order to get an estimate of the mixing ratio of blue, green and red emitting hybrids to yield a white light emitting material upon excitation at 410 nm, a blend of the hybrid materials displaying the second highest quantum yields (**8@MCM-4**, **9@MCM-2**, **10@MCM-2**) was prepared.

**Figure 5:**
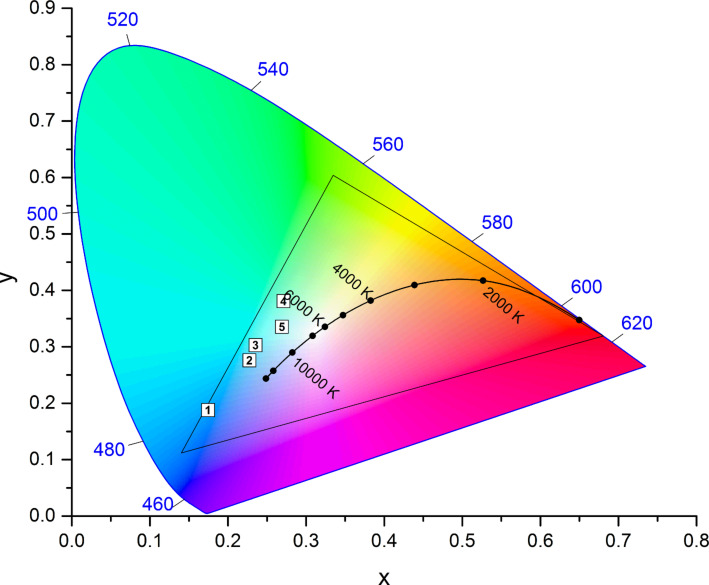
CIE 1931 color space chromaticity diagram (2° observer) with the color space accessible by mixing these three precursors **9** (blue), **10** (green), and **8** (red) in ethanol shown as triangle as well as the CIE coordinates of the hybrid blends **[9@MCM-2 + 10@MCM-2 + 8@MCM-4]-1** to **5**.

**Figure 6:**
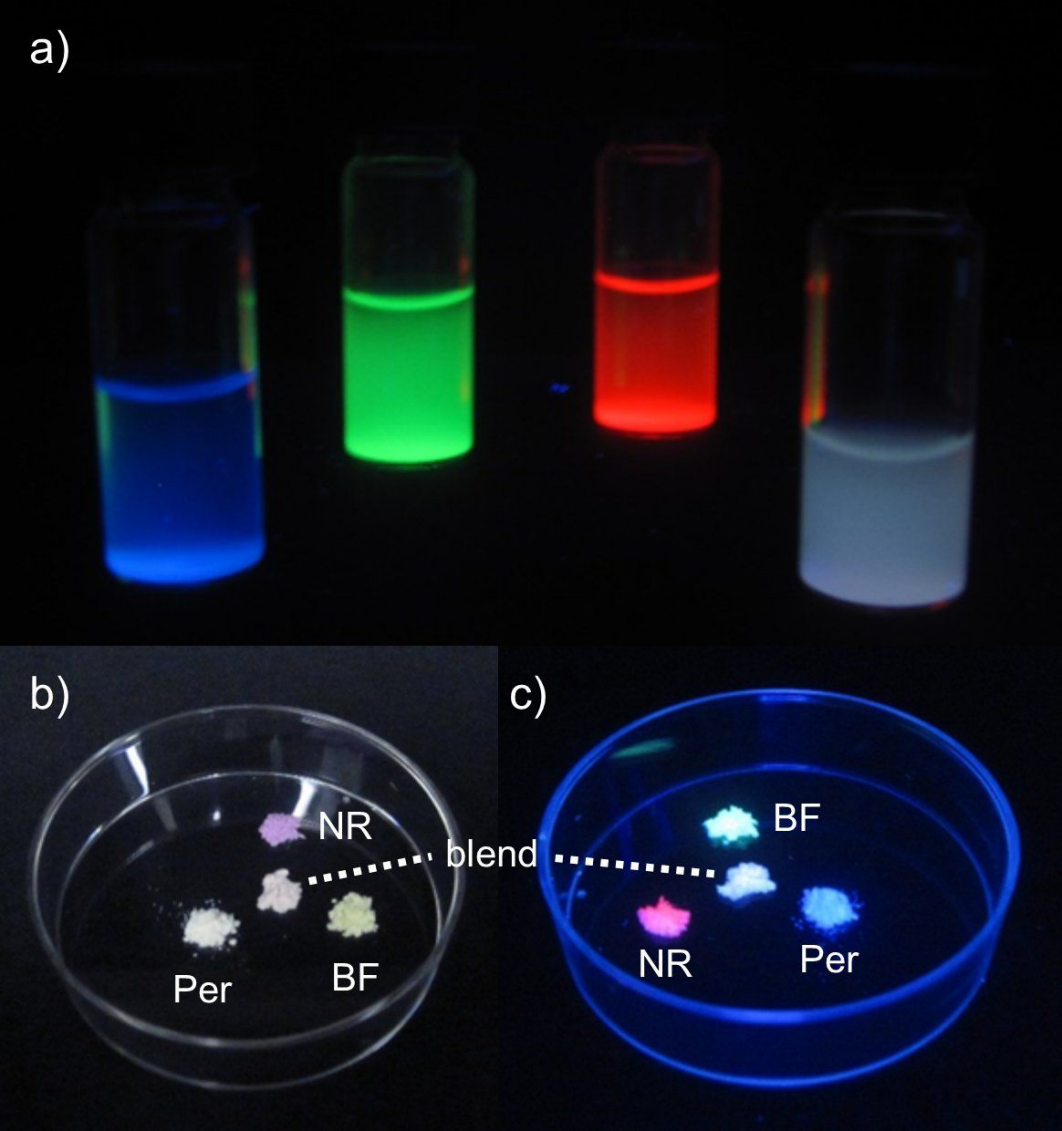
a) Suspensions of the dye-functionalized silica hybrid materials **8@MCM-3**, **9@MCM-3**, and **10@MCM-6** as well as their mixture in dichloromethane under UV light (λ_exc_ = 365 nm). b) Solids of dye-functionalized silica hybrid materials **8@MCM-4**, **9@MCM-2**, and **10@MCM-2** as well as their mixture under daylight. c) Solids of dye-functionalized silica hybrid materials **8@MCM-4**, **9@MCM-2**, and **10@MCM-2** as well as their mixture under UV light (λ_exc_ = 365 nm).

Starting from a blend of **8@MCM-4**, **9@MCM-2**, **10@MCM-2** with a molar ratio of 1:0.1:0.1 the CIE coordinates were determined as x = 0.17 and y = 0.19. Thus, as this first blend was blue emissive and lacked portions of the green and red components, the amount of the green and red hybrid was increased as depicted in Table 2 with their corresponding CIE coordinates shown in Figure 5. Thereby, a blend **[8@MCM-4 + 9@MCM-2 + 10@MCM-2]-5** with CIE coordinates in the proximity of the white point could be obtained. The molar ratio of that blend was used as a starting point for the mixing of the hybrid materials with the highest quantum yields. Thus after determination of the CIE coordinates of a blend of **8@MCM-2**, **9@MCM-3** and **10@MCM-6** with a similar molar ratio, the missing color components could be admixed to yield a white light emitting powder **[8@MCM-2 + 9@MCM-3 +10@MCM-6]-1** with CIE chromaticity coordinates of x = 0.33 and y = 0.33, an external solid state fluorescence quantum yield Φ_f_ of 4.6% and a correlated color temperature of 5500 K (λ_exc_ = 410 nm, molar ratio: 1.0:4.9:1.3). Pictures of a blend of the hybrid materials **8@MCM-4**, **9@MCM-2**, and **10@MCM-2** upon UV excitation (λ_exc_ = 365 nm) in suspension as well as in the solid state are shown in Figure 6.

**Table 2:** Molar ratios of the hybrid blends **[9@MCM-2 + 10@MCM-2 + 8@MCM-4]** with their corresponding CIE coordinates x and y (λ_ex_ = 410 nm).

**hybrid blends**	**9@MCM-2**	**10@MCM-2**	**8@MCM-4**	x	y

**[9@MCM-2 + 10@MCM-2 + 8@MCM-4]-1**	1	0.1	0.1	0.17	0.19
**[9@MCM-2 + 10@MCM-2 + 8@MCM-4]-2**	1	0.5	0.6	0.23	0.28
**[9@MCM-2 + 10@MCM-2 + 8@MCM-4]-3**	1	0.9	0.7	0.24	0.30
**[9@MCM-2 + 10@MCM-2 + 8@MCM-4]-4**	1	2.7	1.4	0.27	0.38
**[9@MCM-2 + 10@MCM-2 + 8@MCM-4]-5**	1	2.7	2.6	0.27	0.34

**Figure 7:**
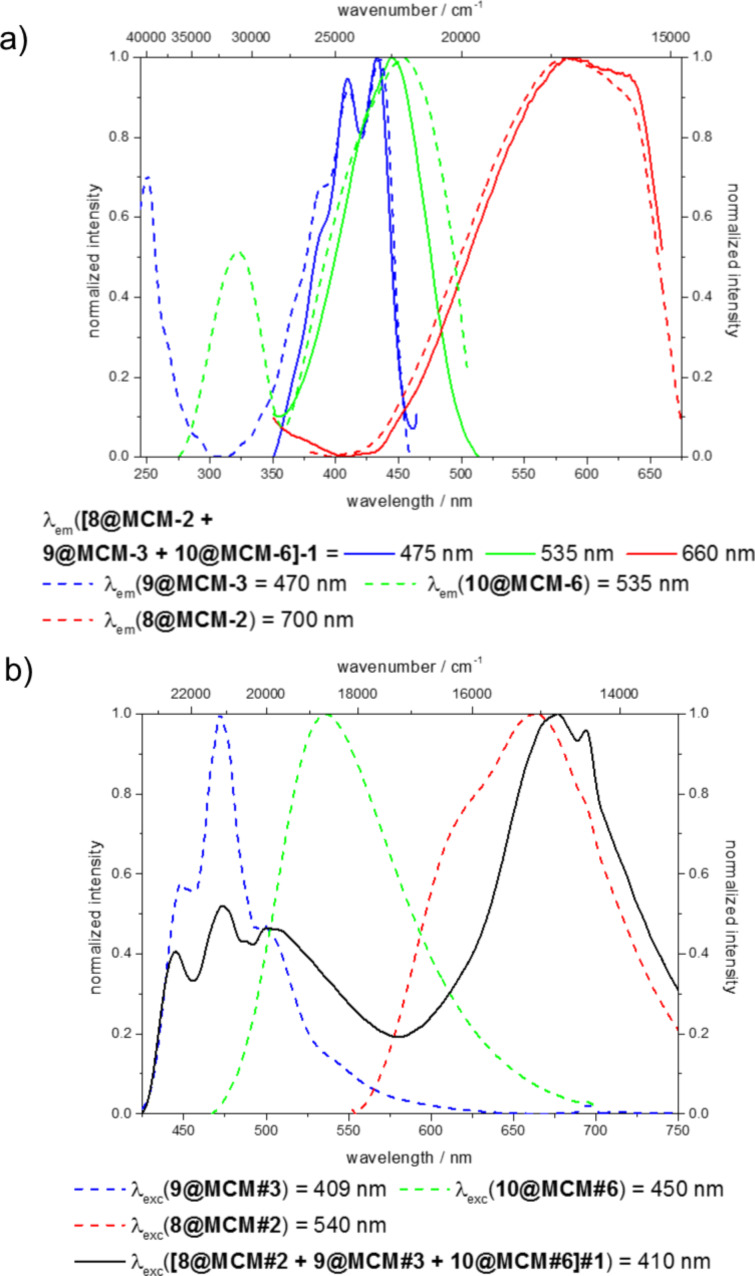
a) Excitation and b) emission spectra of the single dye-functionalized hybrid materials **8@MCM-2**, **9@MCM-3**, and **10@MCM-6** as well as their blend **[8@MCM-2 + 9@MCM-3 + 10@MCM-6]-1**.

Investigation of the excitation and emission spectra of the single dye-functionalized hybrid materials **8@MCM-3**, **9@MCM-3**, and **10@MCM-6** as well as their white light-emitting mixture **[8@MCM-2 + 9@MCM-3 + 10@MCM-6]-1** revealed in first approximation that blending did not alter the spectral appearance in comparison to the constituents, i.e., the recorded spectra are in agreement with an additive spectral behavior (superimposition of the individual spectra of **8@MCM-3**, **9@MCM-3**, and **10@MCM-6**) void of intermolecular energy transfer effects (Figure 7). However, minor differences in the excitation and emission spectra can be found upon comparison of the single dye-functionalized hybrids and their blend, especially for the benzofurazane part.

In the excitation spectra the excitation of the green constituent of the blend **[8@MCM-2 + 9@MCM-3 + 10@MCM-6]-1** detected at an emission wavelength of 535 nm is bathochromically shifted compared to the excitation of the single dye-functionalized material **10@MCM-6**. This dissimilarity arises from the fact that at the emission wavelength of 535 nm not only the benzofurazane-functionalized hybrid **10@MCM-6** shows an emission, but also the perylene-functionalized hybrid **9@MCM-3** (Figure 7b). Thus the excitation spectra at an emission wavelength of 535 nm gives a superimposition of the excitation spectra of the blue and green component in the blend, resulting in a virtual blue shift of the excitation of the green component relative to the pure hybrid **10@MCM-6**.

Similarly, the emission spectra are affected as both the blue and green constituents show emissions in the range of 475–600 nm resulting in a superimposition of both emissions leading to a virtual hypsochromic shift of the emission maximum of the green component of the white light emitting powder **[8@MCM-2 + 9@MCM-3 + 10@MCM-6]-1** relative to the emission spectra of the benzofurazane-functionalized hybrid **10@MCM-6**.

**Figure 8:**
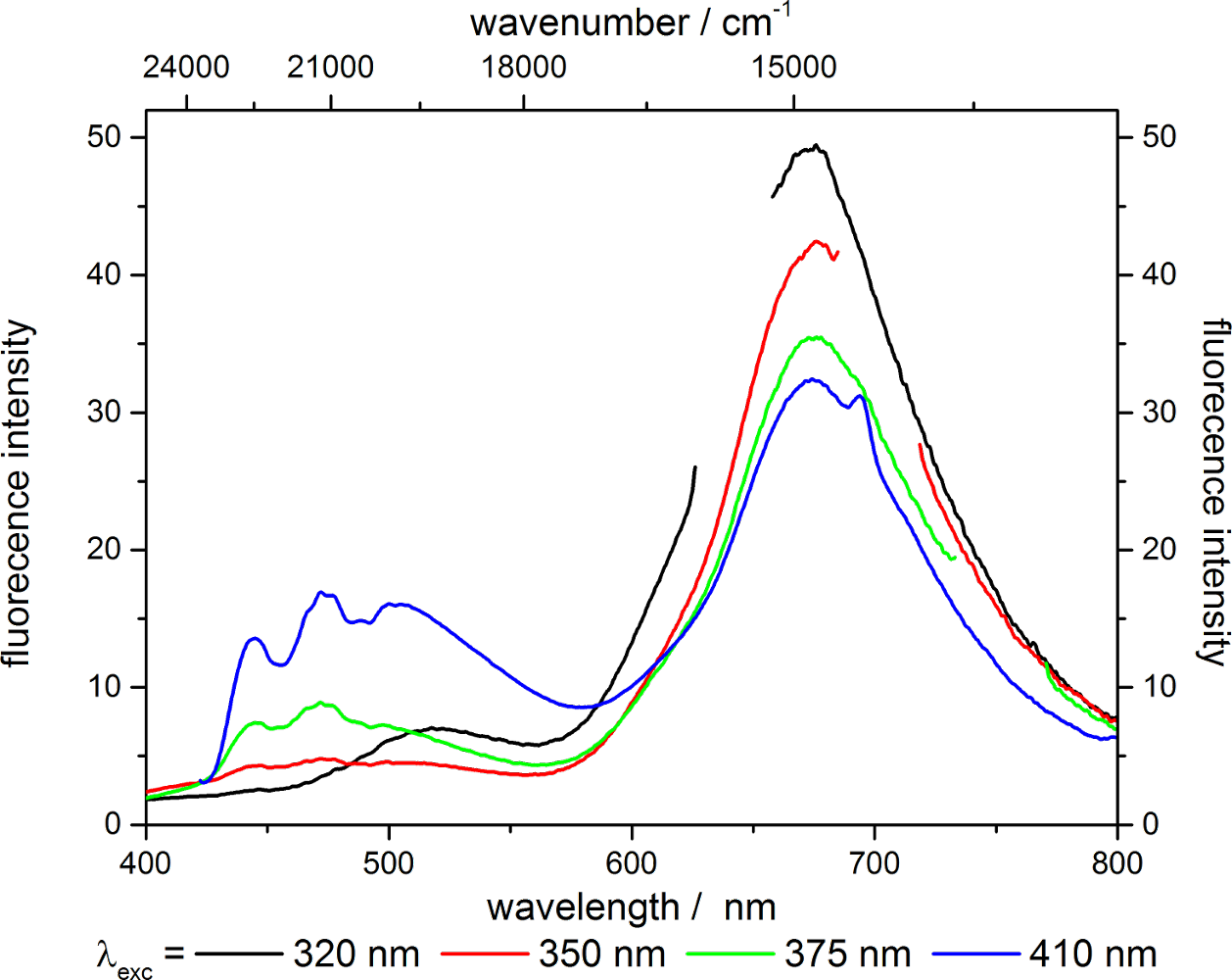
Emission spectra of blend **[8@MCM-2 + 9@MCM-3 + 10@MCM-6]-1** at different excitation wavelengths (2nd order of excitation beam not shown).

Additional information on the excitation mechanism of all three chromophores was gained upon excitation with UV light and recording of emission spectra at different excitation wavelengths (Figure 8).

Upon excitation at 320 nm green as well as intense red emission was found. With increasing excitation wavelength to 350 nm the emissions in the red and green component decrease while the blue component starts to emit. Upon excitation at higher wavelengths, the emissions of the blue and green components increase while the emission intensity of the red component decreases.

These observations are in accordance with the excitation spectra shown in Figure 7a, where only the benzofurazane and Nile red hybrids are excited at 320 nm. With increasing excitation wavelength, the excitation spectra of benzofurazane and Nile red hybrids show a decrease in intensity thus explaining the decrease in fluorescence intensity for the green and red components at an excitation wavelength of 350 nm. Upon further increase of the excitation wavelength the perylene and benzofurazane hybrids show increasing fluorescence intensity as their excitation spectra concomitantly reveal increasing excitation intensity starting from 310 nm for the perylene hybrid and from 355 nm for the benzofurazane material.

As the excitation intensity of the Nile red hybrid decreases up to 400 nm, the decrease in emission intensity shown in Figure 8 is presumably due to this decrease in excitation intensity. These findings thus indicate a direct excitation of all three chromophores in the blend of **[8@MCM-2 + 9@MCM-3 + 10@MCM-6]-1**. Although reabsorption effects are possible, they seem to affect the spectra only to a minor extent, as otherwise an increase of the emission intensity of the red component in Figure 8 should be obtained as the emission intensity of the green component increases. This direct excitation of all three dyes in the blend of **[8@MCM-2 + 9@MCM-3 + 10@MCM-6]-1** also rationalizes the low external solid-state fluorescence quantum yield Φ_f_ of 4.6% as the red component is only poorly excited at 410 nm.

Encouraged by generating white light emitting dye-functionalized silica hybrids upon blending pure dye hybrids we considered incorporating all three dyes simultaneously for the formation of samples of monolithic silica according to literature procedures [17]. For the synthesis of monolithic materials the precursor molecules **8**, **9**, and **10** were mixed in ethanol in a ratio of 1:6:17 furnishing a perfect white emission with the CIE chromaticity coordinates of x = 0.33 and y = 0.33. This mixture was then mixed with TEOS (tetraethoxysilane), surfactant P123 (poly(ethylene glycol)-poly(propylene glycol)-poly(ethylene glycol/(EtO)_20_(iPrO)_70_(EtO)_20_), HCl and water. After 36 h of aging at room temperature, the samples were covered with *n*-octane and aged at 60 °C for a period of 1–4 d, depending on the shape of the reaction vessel, until the samples were dry.

**Figure 9:**
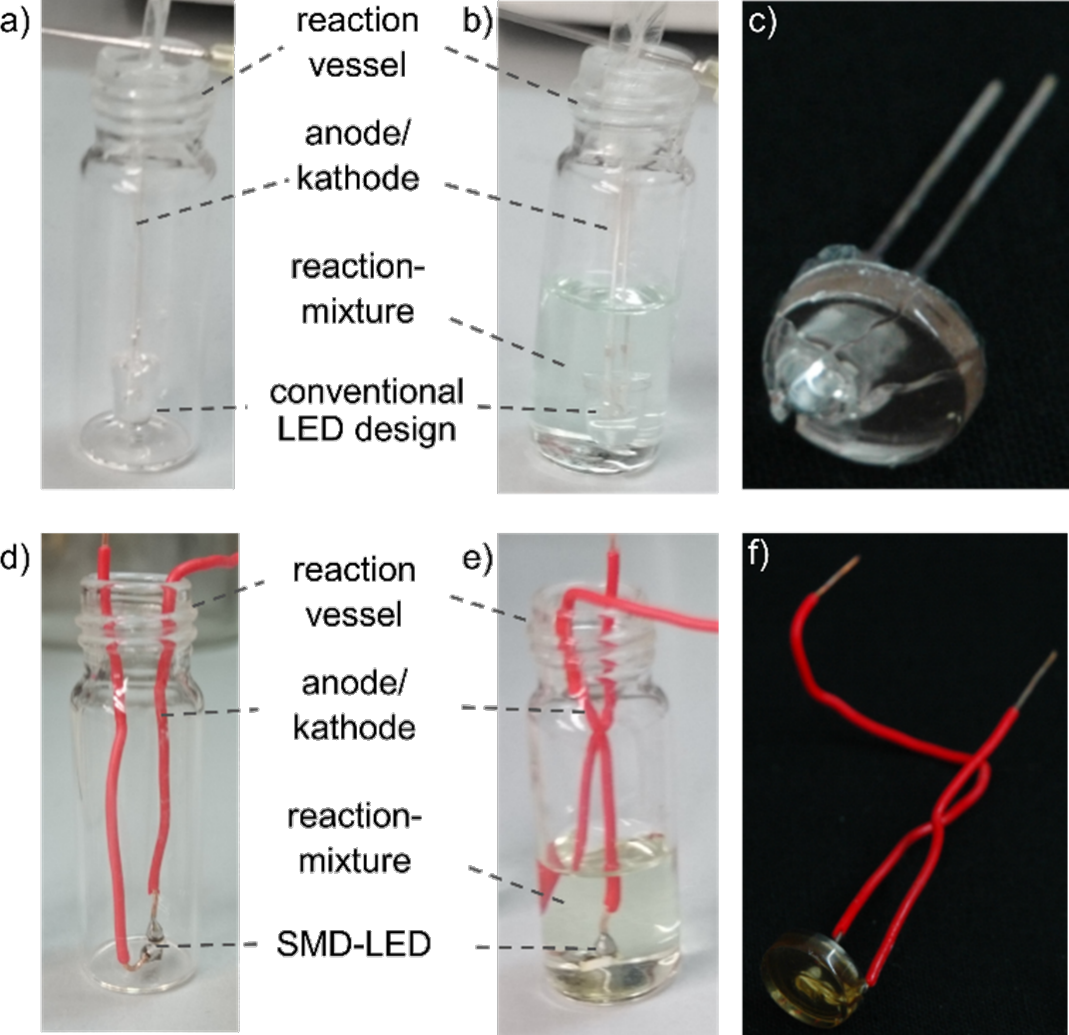
Coating of the a) conventional diode setup and b) surface-mounted device (SMD) (left: prior to the addition of the reaction mixture, right: after the addition), c) coated conventional diode setup, d) coated surface-mounted device.

With this formation of white light emitting monoliths, as a proof of concept, we prepared four examples of white light emitting LEDs with organic phosphors. Therefore, UV-emitting LEDs were early immersed into the reaction mixture upon monolith formation. After aging, according to the described method, the UV emitting diodes were coated with the white light emitting hybrid materials as shown in Figure 9. Two different LED designs were investigated, a conventional diode setup as well as a much smaller surface mounted device (SMD).

**Figure 10:**
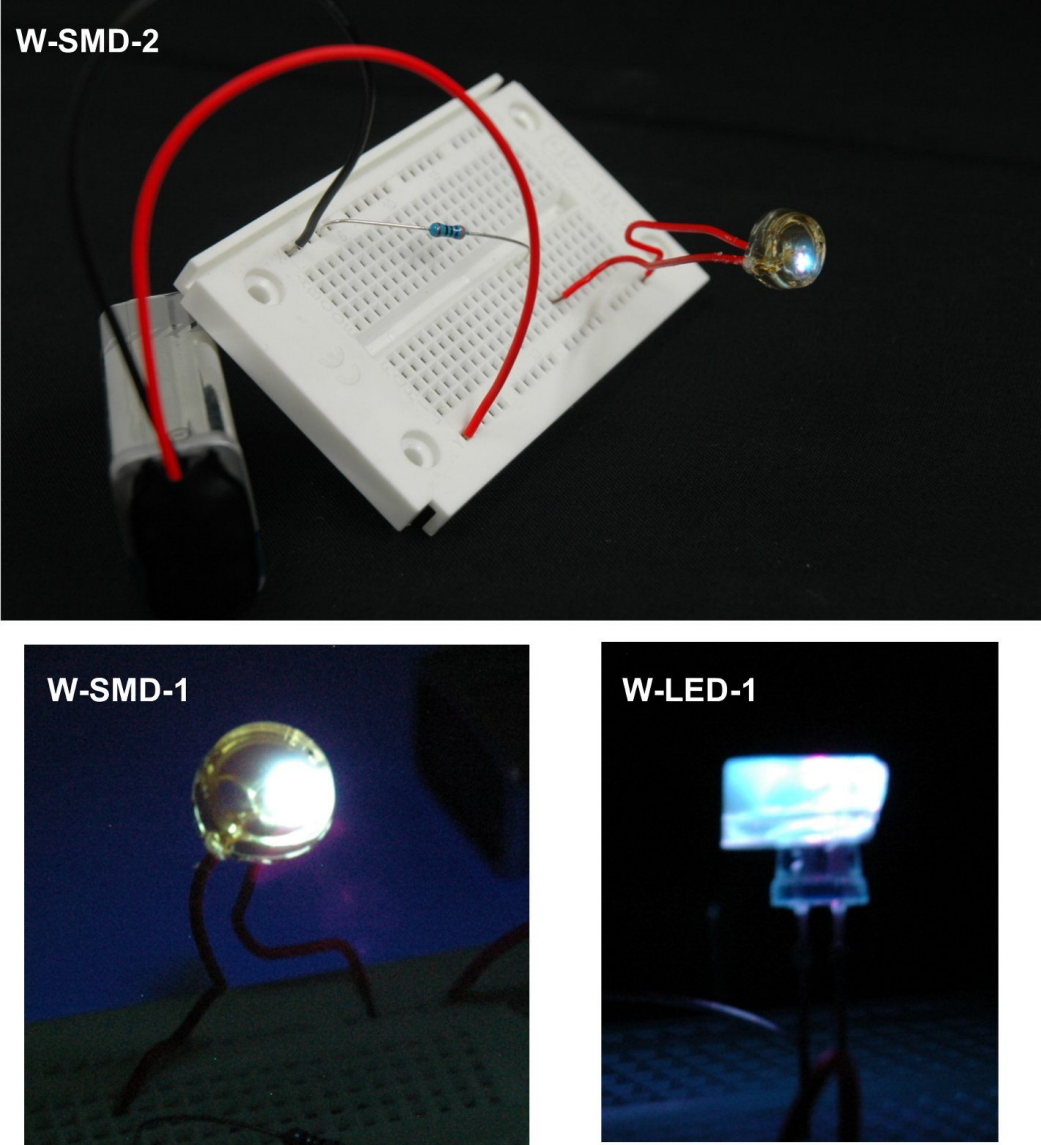
Pictures of the coated LEDs in compact device set-up (SMD) and conventional diode design (LED).

**Figure 11:**
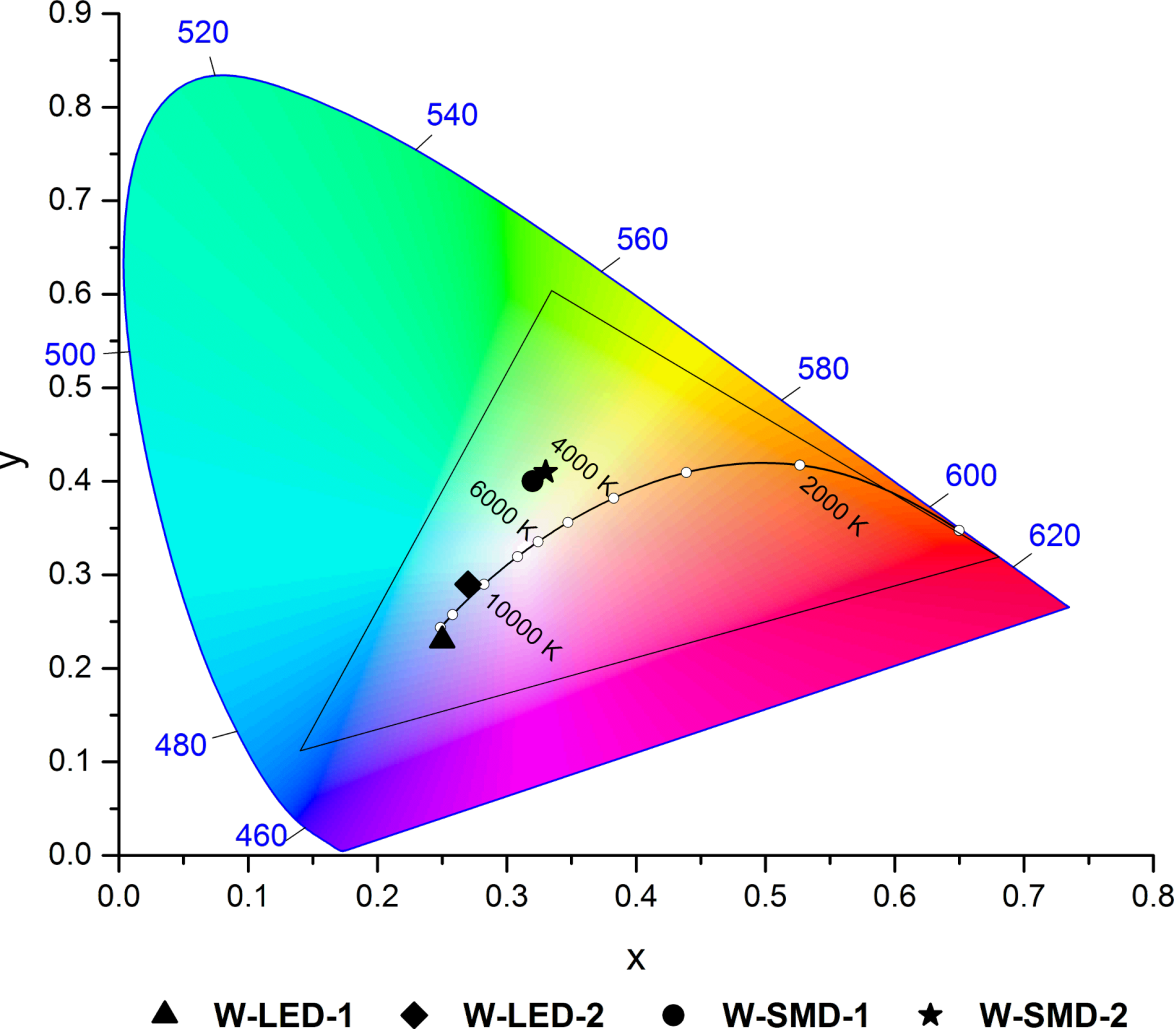
CIE chromaticity coordinates of the coated LEDs in compact device set-up (SMD) and conventional diode design (LED) in the CIE 1931 color space (2° standard observer).

These coated LEDs were spectroscopically analyzed displaying correlated color temperatures of 10700 K and 41100 K for the conventional LED setup, overlapping with the spectrum of the black body radiator. For the SMD setup warmer color temperatures were obtained, but their CIE chromaticity coordinates are slightly shifted from the emission of the black body radiator (Table 3, Figure 10, Figure 11).

**Table 3:** CIE-coordinates x and y and correlated color temperatures of the four monolith coated LEDs.

Sample	x	y	correlated color temperature [K]

**W-LED-1**	0.25	0.23	41104
**W-LED-2**	0.27	0.29	10686
**W-SMD-1**	0.32	0.40	5785
**W-SMD-2**	0.33	0.41	5530

## Conclusion

In summary, we were able to synthesize triethoxysilylpropyl-substituted perylene, benzofurazane, and Nile red precursor molecules and to incorporate them into sol–gel derived silica matrices. This was achieved by postsynthetic grafting of commercially available MCM-41 yielding single dye-functionalized powders, which could be mixed to give white light-emitting powders upon excitation with UV light. Furthermore, commercially available UV emitting diodes were coated with silica monoliths composed of a mixture of the perylene, benzofurazane and Nile red precursor molecules serving as UV light converting phosphors to yield white light emission without employing of inorganic luminophores. Further optimization of the dye loadings of these monoliths as well as fine tuning of the correlated color temperatures and determination of their luminescence efficiencies is currently under way. Additionally, studies on the photostability of the hybrid materials are in the focus of further research. Further investigations are directed to the determination of quantum yields of the monolith-functionalized materials since their quantum yields could show higher efficiencies in comparison to the powder samples due to elimination of scattering of the excitation and emission light in the silica particles.

## Supporting Information

File 1 Multistep synthetic procedures, spectroscopic and analytical data of the precursors **9** and **10**, and the syntheses of the dye-functionalized MCM-41 hybrid materials **8@MCM**, **9@MCM**, and **10@MCM** as well as the determined dye loadings of the hybrids.
