# Supplementary material for: Energy down converting organic fluorophore functionalized mesoporous silica hybrids for monolith-coated light emitting diodes
Source: Beilstein J Org Chem. 2017 Apr 25;13:768–78. doi: 10.3762/bjoc.13.76 (PMC5433209; doi:10.3762/bjoc.13.76)
Supplement: File 1 — Multistep synthetic procedures, spectroscopic and analytical data of the precursors 9 and 10, and the syntheses of the dye-functionalized MCM-41 hybrid materials 8@MCM, 9@MCM, and 10@MCM as well as the determined dye loadings of the hybrids. [file Beilstein_J_Org_Chem-13-768-s001.pdf]

Supporting Information

for

Energy down converting organic fluorophore functionalized mesoporous silica hybrids for monolith-coated light emitting diodes

Markus Börgardts and Thomas J. J. Müller*

Address: Institut für Organische Chemie und Makromolekulare Chemie, Heinrich-Heine-Universität Düsseldorf, Universitätsstr. 1, D-40225 Düsseldorf, Germany

Email: Thomas J. J. Müller - ThomasJJ.Mueller@uni-duesseldorf.de

* Corresponding author

Multistep synthetic procedures, spectroscopic and analytical data of the precursors **9** and **10**, and the syntheses of the dye-functionalized MCM-41 hybrid materials **8@MCM**, **9@MCM**, and **10@MCM** as well as the determined dye loadings of the hybrids

Table of Contents

1. General considerations S3

2. Synthetic procedures S4

2.1. Precursors S4

2.1.1. 3-Formylperylene [2] S4

2.1.2. Perylen-3-ylmethanol (2) [2] S4

2.1.3. 3-[(Prop-2-yn-1-yloxy)methyl]perylene (5) [3] S5

2.1.4. 4-((Perylene-3-ylmethoxy)methyl)-1-(3-(triethoxysilyl)propanyl)-1H-1,2,3-triazol (9) S6

2.1.5. 1,3-Dichloro-2-nitrobenzene [4] S7

2.1.6. 4-Chlorobenz[c][1,2,5]oxadiazole [4] S7

2.1.7. 4-Chloro-7-nitrobenz[c][1,2,5]oxadiazole (3) [4] S8

2.1.8. 7-Nitro-*N*-(prop-2-yn-1-yl)benz[*c*][1,2,5]oxadiazole-4-amine (6) [4] S8

2.1.9. 7-Nitro-*N*-((1-(3-(triethoxysilyl)propyl)-1*H*-1,2,3-triazol-4-yl)methyl)benzo[*c*][1,2,5]oxadiazole-4-amine (10) S9

2.2. Synthesis of the dye-functionalized MCM-41 hybrid materials S10

2.2.1. Synthesis of Nile red-functionalized hybrid materials 8@MCM S10

2.2.2. Synthesis of perylene and benzofurazane-functionalized hybrid materials 9@MCM and 10@MCM S11

1. General considerations

Reagents, catalyst and solvents were purchased reagent grade and used without further purification. Products were purified with column chromatography on silica gel 60 (0.040–0.063 mm) from M&N using flash technique.

^{1}H, ^{13}C and 135-DEPT NMR spectra were recorded on a Bruker AVIII-300 and the resonance of the particular solvent was locked as internal standard ($CDCl_3$: ^{1}H δ = 7.26, ^{13}C δ = 77.0, DMSO-d_6: ^{1}H δ = 2.50, ^{13}C δ = 39.52). MALDI mass spectra were measured on a Bruker Daltronics Ultraflex I spectrometer, HR-ESI mass spectra were measured on a UHR-QTOF maXis 4G Bruker Daltronics and GC–mass spectra were obtained on a GCMS-QP2010 S spectrometer from Shimadzu. Combustion analyses were carried out on Elementar vario MICRO CUBE in the microanalytical laboratory of the Institut für Pharmazeutische und Medizinische Chemie at the Heinrich-Heine-Universität Düsseldorf. IR spectra were recorded on Shimadzu IRAffinity-1. Melting points were measured on a Büchi Melting Point B-540. N_2-adsorption-desorption isotherms were determined at 77 K by a Quantachrome Nova 4200e sorption analyser. Prior to the measurements the samples were degassed at 80.0 °C for 20 h. Specific surface areas were calculated using the Brunauer–Emmett–Teller (BET) equation in the low pressure interval $p/p_0 < 0.3$. DFT pore size distributions were calculated from the adsorption branch and the total pore volume was calculated at the point of $p/p_0 = 0.95$. X-ray diffraction data were collected on a Bruker AXS Nanostar C. The radiation source was a Siemens X-ray canal with a power of 1500 W. The nickel-filtered monochromatic Cu Kα1 radiation wavelength of 1.5405 nm was maintained by using crossed Göbel mirrors as monochromator. Real-time detection was enabled using a Bruker HI-Star detector. Samples were measured in Hilgenberg glass capillaries with an outer diameter of 0.7 nm. Pre-measurement calibration was carried out at 298 K with silver behenate as a standard reference. Data analysis was carried out using the following software: SAXS from Bruker, Datasqueeze (v. 2.2.8) from Heiney, QTIPlot (v.0.9.8) from Ion Vasilief and LCDiXray (v.1.0) from Golbert. Mesoporous structures were probed by HR-TEM at the institute of microstructure research in Jülich using a FEI TECNAI G^2 operated at 200 kV. All HR-TEM samples were prepared by placing a drop of the hybrid material suspension in dichloromethane onto a carbon-coated grid at room temperature.

Excitation and emission spectra were recorded on a Hitachi F-7000 fluorescence spectrophotometer at T = 293 K. Excitation of fluorescence was always carried out at the excitation maximum. Quantum yield determinations of the hybrid powders were obtained with an integrating sphere. Data analysis and quantum yield calculations were performed with the software FL Solutions Version 4.0 by Hitachi.

2. Synthetic procedures

2.1. Precursors

Nile red derivatives **1**, **4**, and **8** and (3-azidopropyl)triethoxysilane (**7**) were prepared according to our previously published procedures [1].

2.1.1. 3-Formylperylene [2]

Perylene (981 mg, 3.89 mmol) was dissolved in 1,2-dichlorobenzene (2 mL). Then DMF (1.85 g, 25.3 mmol) was added and the solution was heated to 100 °C. Over a period of 10 min phosphorus trichloride (1.19 g, 7.78 mmol) was added dropwise and the reaction mixture was heated to 100 °C for 3.5 h. Thereafter the solution was poured into 150 mL of an aqueous sodium acetate solution and cooled to 0 °C for 3 h. The precipitate was filtered off, adsorbed to Celite and purified by chromatography on silica gel ($CHCl_3$) to give 3-formylperylene (480 mg, 1.71 mmol, 44%) as an orange solid.

Mp 218-224 °C.

R_f (*n*-hexane/ethyl acetate 50:1) = 0.16.

^{1}H NMR (300 MHz, $CDCl_3$): δ = 7.38 - 7.45 (m, 2 H), 7.59 (dd, ^3J = 7.7 Hz, ^3J = 8.4 Hz, 1 H), 7.69 (m, 1 H), 7.75 (m, 1 H), 7.79 (d, ^3J = 7.9 Hz, 1 H), 8.09 - 8.20 (m, 4 H), 9.07 (dd, ^3J = 8.5, ^4J = 1.0, 1 H), 10.23 (s, 1 H).

^{13}C NMR (75 MHz, $CDCl_3$): δ = 119.0 (CH), 121.0 (CH), 121.5 (CH), 122.7 (CH), 124.6 (CH), 126.6 (CH), 126.9 (CH), 128.1 (C_{quat}), 128.5 (CH), 128.9 (C_{quat}), 129.2 (CH), 129.8 (C_{quat}), 129.9 (C_{quat}), 130.0 (CH), 130.6 (C_{quat}), 131.2 (C_{quat}), 132.2 (C_{quat}), 134.3 (C_{quat}), 137.1 (CH), 137.6 (C_{quat}), 192.7 (CH).

ESI: *m*/*z* = 281.1 $[C_{21}H_{13}O + H]^+$.

IR (ATR): $\tilde{\nu}$ / cm^{-1} = 644 m, 664 w, 704 w, 741 vs, 760 vs, 808 vs, 824 s, 895 m, 962 w, 978 w, 1030 m, 1051 m, 1084 s, 1132 m, 1157 s, 1190 m, 1211 s, 1225 m, 1283 m, 1308 w, 1337 w, 1360 w, 1385 m, 1408 m, 1433 m, 1454 m, 1503 s, 1518 s, 1564 s, 1587 m, 1636 w, 1651 w, 1680 s, 1732 m, 1790 w, 1867 w, 1938 w, 2093 w, 2351 w, 2710 w, 2724 w, 2874 w, 2901 m, 2922 m, 2959 m, 3048 w, 3098 w, 3345 w, 3647 w, 3686 w.

Anal. calcd. for $C_{21}H_{12}O$ (280.3): C 89.98, H 4.31; Found: C 89.70, H 4.41.

2.1.2. Perylen-3-ylmethanol (2) [2]

3-Formylperylene (93.0 mg, 0.332 mmol) was dissolved in dry THF (25 mL) before a mixture of lithium aluminum hydride (35.3 mg, 0.664 mmol) in THF (5 mL) was added. After stirring at room temperature for 30 min deionised water (30 mL) and $CHCl_3$ (50 mL) were added to the reaction mixture. The organic layer was separated and the aqueous layer washed with $CHCl_3$ (3 × 50 mL). The combined organic layers were dried with anhydrous magnesium sulfate and the solvents were removed in vacuo. The residue was adsorbed on Celite and purified by

chromatography on silica gel ($CHCl_3$) to give perylen-3-ylmethanol (**2**, 61.0 mg, 0.216 mmol, 65%) as a yellow solid.

Mp 204-209 °C.

R_f ($CHCl_3$) = 0.32.

^{1}H NMR (300 MHz, $CDCl_3$): δ = 5.10 (s, 2 H), 7.46-7.59 (m, 4 H), 7.70 (dd, ^3J = 8.4 Hz, ^4J = 0.7 Hz, 2 H), 7.96 (dd, ^3J = 8.4 Hz, ^4J = 0.9 Hz, 1 H), 8.15-8.26 (m, 4 H).

^{13}C NMR (75 MHz, $CDCl_3$): δ = 64.0 (CH_2), 119.9 (CH), 120.5 (CH), 120.6 (CH), 123.6 (CH), 126.2 (CH), 126.8 (CH), 127.0 (CH), 128.1 (CH), 128.6 (C_{quat}), 129.2 (C_{quat}), 131.2 (C_{quat}), 131.4 (C_{quat}), 131.7 (C_{quat}), 131.9 (C_{quat}), 132.7 (C_{quat}), 134.8 (C_{quat}), 134.0 (C_{quat}).

ESI: m/z = 282.1 $[C_{21}H_{14}O]^+$.

IR (ATR): $\tilde{\nu}$ / cm^{-1} = 748 m, 762 vs, 785 w, 804 m, 822 m, 872 w, 901 w, 937 w, 962 w, 991 m, 1043 w, 1059 m, 1082 m, 1132 w, 1155 w, 1186 w, 1236 w, 1287 w, 1387 m, 1503 m, 1591 m, 2920 m, 2961 m, 3045 w, 3264 br.

2.1.3. 3-[(Prop-2-yn-1-yloxy)methyl]perylene (5) [3]

Perylen-3-ylmethanol (**2**, 150 mg, 0.532 mmol) and 18-crown-6 were dissolved in THF (10 mL) before a solution of potassium hydroxide (6.66 g, 119 mmol) in deionised water (6.66 g) was added. After stirring for 30 min at room temperature propargyl bromide (325 mg, 2.73 mmol) was added and the reaction mixture was stirred for 1 h at room temperature. The solvent was removed in vacuo and the residue was dissolved in 50 mL $CHCl_3$ and 50 mL deionised water. The organic layer was separated and the aqueous layer was extracted with $CHCl_3$ (3 × 50 mL). The combined organic layers were dried with magnesium sulfate and the solvents were removed in vacuo. The residue was adsorbed on Celite and purified by chromatography on silica gel (*n*-hexane/ethyl acetate 5:1) to give 3-[(prop-2-yn-1-yloxy)methyl]perylene (**5**) (57.6 mg, 0.189 mmol, 33%) as a yellow solid.

Mp 152-161 °C.

R_f (*n*-hexane/ethyl acetate 5:1) = 0.55.

^{1}H NMR (300 MHz, $CDCl_3$): δ = 2.54 (t, ^4J = 2.4 Hz, 1 H), 4.26 (d, ^4J = 2.4 Hz, 2 H), 5.00 (s, 2H), 7.45-7.58 (m, 4 H), 7.66-7.71 (m, 2 H), 7.98 (dd, ^3J = 8.4 Hz, ^4J = 1.0 Hz, 1 H), 8.11-8.25 (m, 4 H).

^{13}C NMR (75 MHz, $CDCl_3$): δ = 57.2 (CH_2), 70.1 (CH_2), 75.0 (CH), 79.8 (C), 119.7 (CH), 120.5 (CH), 124.0 (CH), 126.7 (CH), 127.0 (CH), 128.0 (CH), 128.1 (CH), 128.6 (C_{quat}), 129.2 (C_{quat}), 131.1 (C_{quat}), 131.3 (C_{quat}), 131.7 (C_{quat}), 132.0 (C_{quat}), 132.4 (C_{quat}), 133.3 (C_{quat}), 134.7 (C_{quat}).

MALDI: m/z = 320.1 $[C_{24}H_{16}O + H]^+$.

IR (ATR): $\tilde{\nu}$ / cm^{-1} = 664 s, 696 m, 737 m, 758 s, 767 vs, 810 vs, 824 vs, 876 m, 891 m, 930 w, 968 w, 1018 m, 1057 vs, 1159 w, 1188 w, 1211 w, 1234 w, 1288 w, 1321 w, 1348 w, 1389

w, 1456 w, 1501 w, 1589 w, 1734 w, 1879 w, 1931 w, 2118 w, 2361 w, 2849 w, 2870 w, 2895 w, 2924 w, 2951 w, 3046 w, 3071 w, 3281 s.

2.1.4. 4-((Perylene-3-ylmethoxy)methyl)-1-(3-(triethoxysilyl)propanyl)-1*H*-1,2,3-triazol (9)

3-[(Prop-2-yn-1-yloxy)methyl]perylene (**5**, 250 mg, 0.781 mmol), copper iodide (222 mg, 1.17 mmol) were dissolved in dry THF (23 mL). Then a mixture of diisopropylethylamine (290 mg, 1.17 mmol) and (3-azidopropyl)triethoxysilane (290 mg, 1.17 mmol) was added and the mixture was stirred at 60 °C for 21 h. Thereafter, deionised water (80 mL) was added to the reaction mixture and the aqueous layer was extracted with dichloromethane (4 × 40 mL). The combined organic layers were dried with anhydrous magnesium sulfate and the solvent was removed in vacuo. The residue was purified by chromatography on silica gel (*n*-hexane/ethyl acetate 1:1) to give 4-((perylene-3-ylmethoxy)methyl)-1-(3-(triethoxysilyl)propanyl)-1*H*-1,2,3-triazol (**9**, 193 mg, 0.349 mmol, 44%) as a yellow powder.

Mp 73-77 °C.

^{1}H NMR (300 MHz, CD_2Cl_2): δ = 0.55-0.62 (m, 2 H), 1.18 (t, ^{3}J = 7.0 Hz, 9 H), 1.99 (m, 2 H), 3.78 (q, ^{3}J = 7.0 Hz, 6 H), 4.33 (t, ^{3}J = 7.2 Hz, 2 H), 4.75 (m, 2 H), 4.97 (m, 2 H), 7.54 (m, 5 H), 7.71 (m, 2 H), 7.93 (dd, ^{3}J = 8.4 Hz, ^{4}J = 1.0 Hz, 1 H), 8.23 (m, 4 H).

^{13}C NMR (75 MHz, CD_2Cl_2): δ = 7.8 (CH_2), 18.5 (CH_3), 24.6 (CH_2), 30.1 (CH_2), 58.8 (CH_2), 64.1 (CH_2), 71.1 (CH_2), 120.0 (CH), 120.7 (CH), 120.8 (CH), 123.1 (CH), 124.4 (CH), 127.0 (CH), 127.1 (CH), 127.8 (CH), 128.3 (CH), 128.8 (C_{quat}), 131.4 (C_{quat}), 131.5 (C_{quat}), 131.8 (C_{quat}), 131.9 (C_{quat}), 133.4 (C_{quat}), 133.8 (C_{quat}), 134.0 (C_{quat}), 135.0 (C_{quat}), 145.2 (C_{quat}).

MALDI: *m*/*z* = 568.2 $[C_{33}H_{38}N_3O_4Si + H]^+$.

IR (ATR): $\tilde{\nu}$ / cm^{-1} = 736 m, 756 vs, 773 vs, 785 m, 814 vs, 829 s, 934 m, 949 s, 1053 s, 1074 vs, 1099 s, 1159 m, 1188 w, 1213 w, 1288 w, 1348 w, 1366 w, 1389 m, 1441 w, 1456 w, 1467 w, 1501 w, 1589 w, 2884 m, 2926 m, 2972 m, 3047 w.

Anal. calcd. for $C_{33}H_{37}N_3O_4Si$ (567.8): C 69.81, H 6.57, N 7.40; Found: C 69.74, H 6.36, N 7.02.

2.1.5. 1,3-Dichloro-2-nitrobenzene [4]

A solution of 2,6-dichloroaniline (8.10 g, 50.0 mmol) in dichloromethane (200 mL) was cooled to 0 °C before a solution of 3-chloroperbenzoic acid (24.6 g, 100 mmol) in dichloromethane (250 mL) was added dropwise to the reaction mixture over a period of 1.5 h at 0 °C. Thereafter the solution was stirred for 2 h at room temperature before it was diluted with dichloromethane (250 mL). The reaction mixture was washed two times with an aqueous sodium thiosulfate solution (2 wt %, 2 × 100 mL), with an aqueous sodium bicarbonate solution (4 × 50 mL), and finally with deionized water (200 mL). The organic layer was dried with magnesium sulfate and the solvent was removed in vacuo. The residue was suspended in 40 mL *n*-hexane and irradiated by ultrasound for 5 min, filtered off, washed with *n*-hexane (2 × 20 mL) to give 1,3-dichloro-2-nitrobenzene (6.63 g, 37.7 mmol, 75%) as a beige solid.

Mp 165-177 °C.

^{1}H NMR (300 MHz, $CDCl_3$): δ = 7.39-7.54 (m).

EI: *m*/*z* = 178.9 ([$^{37}Cl_2$-M]$^+$, 12), 176.9 ([$^{37}Cl^{35}Cl$-M]$^+$, 66.5), 175.0 ([$^{35}Cl_2$-M]$^+$, 100).

IR (ATR): $\tilde{\nu}$ / cm^{-1} = 640 s, 667 s, 719 vs, 748 vs, 781 vs, 853 s, 880 s, 899 s, 914 m, 1076 m, 1103 m, 1140 m, 1188 w, 1207 s, 1263 s, 1287 s, 1418 s, 1568 s, 1682 s, 2552 w, 2598 w, 2664 w, 2710 w, 2813 w, 2884 w, 2965 w, 3076 w.

2.1.6. 4-Chlorobenzo[*c*][1,2,5]oxadiazole [4]

To a solution of 1,3-dichloro-2-nitrobenzene (6.59 g, 37.4 mmol) in DMSO (150 mL) sodium azide (2.43 g, 37.4 mmol) was added over a period of 30 min. The reaction mixture was stirred at room temperature for 2.5 h until the evolution of gas subsided. Thereafter, the solution was heated to 120 °C for 35 min. After addition of deionized water (300 mL) the mixture was extracted with diethyl ether (4 × 225 mL). The combined organic layers were dried with anhydrous magnesium sulfate and the solvent was removed in vacuo. The residue was dissolved in refluxing ethanol (80 mL), whereupon deionized water (80 mL) was added and a precipitate was formed. The precipitate was filtered off and the filtrate was diluted with deionized water (250 mL) upon which a second precipitate was formed. The combined precipitates were dried in vacuo to give 4-chlorobenzo[*c*][1,2,5]oxadiazole (4.69 g, 30.3 mmol, 81%) as an orange-brown solid.

Mp 100-102 °C.

R_f (*n*-hexane/ethyl acetate 1:1) = 0.62.

^{1}H NMR (300 MHz, $CDCl_3$): δ = 7.32-7.47 (m, 2 H), 7.77 (dd, ^{3}J = 8.8 Hz, ^{4}J = 0.7 Hz, 1 H).

^{13}C NMR (75 MHz, $CDCl_3$): δ = 115 (CH), 123 (C_{quat}), 130 (CH), 132 (CH), 149 (C_{quat}), 150 (C_{quat}).

EI: *m*/*z* = 156 ([^{37}Cl-M]$^+$, 31), 154 ([^{35}Cl-M]$^+$, 100).

IR (ATR): $\tilde{\nu}$ / cm^{-1} = 627 s, 745 s, 812 m, 835 m, 866 s, 883 s, 914 m, 961 s, 980 m, 1018 m, 1030 s, 1227 m, 1339 m, 1368 m, 1395 m, 1445 m, 1526 s, 1616 m, 3379 br.

2.1.7. 4-Chloro-7-nitrobenzo[*c*][1,2,5]oxadiazole (3) [4]

A solution of $NaNO_3$ (14.6 g, 172 mmol) in H_2SO_4 (250 g, 2.55 mol) was cooled to 0 °C before 4-chlorbenzo[*c*][1,2,5]oxadiazole (4.59 g, 29.6 mmol) was added to the solution. After stirring for 5 min at 0 °C the reaction mixture was heated to 85 °C for 30 min. Thereafter ice water (250 mL) was added and cooled to 0 °C. The precipitate was filtered off and recrystallized from an ethanol water mixture (1.6:1, 400 mL) to give 4-chloro-7-nitrobenzo[*c*][1,2,5]oxadiazole (**3**, 3.22 g, 16.1 mmol, 54%) as yellow needles.

Mp 100-102 °C.

R_f (*n*-hexane/ethyl acetate 1:1) = 0.30.

^{1}H NMR (300 MHz, $CDCl_3$): δ = 7.64-7.69 (m, 1 H), 8.45-8.50 (m, 1 H).

^{13}C NMR (75 MHz, $CDCl_3$): δ = 129 (CH), 130 (CH), 131 (C_{quat}), 136 (C_{quat}), 143 (C_{quat}), 149 (C_{quat}).

EI: *m*/*z* = 200.9 ([^{37}Cl-M]$^+$, 32), 198.9 ([^{35}Cl-M]$^+$, 100),

IR (ATR): $\tilde{\nu}$ / cm^{-1} = 677 s, 731 s, 773 s, 808 s, 864 s, 895 s, 964 s, 1005 s, 1051 s, 1091 w, 1121 w, 1217 m, 1254 m, 1296 m, 1310 s, 1325 s, 1366 s, 1451 s, 1518 s, 1632 m, 1921 w, 3098 m.

2.1.8. 7-Nitro-*N*-(prop-2-yn-1-yl)benzo[*c*][1,2,5]oxadiazole-4-amine (6) [4]

To a stirred suspension of 4-chloro-7-nitrobenzo[*c*][1,2,5]oxadiazole (**3**) (1.51 g, 7.55 mmol) in a THF/EtOH mixture (1:1, 5 mL), sodium bicarbonate (3.36 g, 40.0 mmol) and propargyl amine (551 mg, 10.0 mmol) were subsequently added. After stirring at room temperature for 80 min, deionized water (500 mL) was added to the reaction mixture and the aqueous layer was extracted with dichloromethane (5 × 200 mL). The combined organic layers were dried with anhydrous magnesium sulfate and the solvent was removed in vacuo. The residue was adsorbed to celite and purified by chromatography on silica gel (*n*-hexane/ethyl acetate 2:1) to give 7-nitro-*N*-(prop-2-yn-1-yl)benzo[*c*][1,2,5]oxadiazole-4-amine (**6**, 986 mg, 4.52 mmol, 45%) as an orange solid.

Mp 150-152 °C.

R_f (*n*-hexane/ethyl acetate 2:1) = 0.58.

^{1}H NMR (300 MHz, acetone-d_6): δ = 2.77-2.81 (m, 1 H), 4.32-4.39 (m, 2 H), 6.59 (d, ^3J = 8.7 Hz, 1 H), 8.45 (br, 1 H), 8.62 (d, ^3J = 8.7 Hz, 1 H).

^{13}C NMR (75 MHz, acetone-d_6): δ = 33.5 (CH_2), 74.3 (C_{quat}), 78.8 (C_{quat}), 101.1 (CH), 125.0 (C_{quat}), 137.5 (CH), 144.5 (C_{quat}), 145.0 (C_{quat}), 145.6 (C_{quat}).

EI: *m*/*z* = 219.0 (14), 217.9 ([M]$^+$, 100),

IR(ATR): $\tilde{\nu}$ / cm^{-1} = 642 s, 656 m, 691 s, 702 s, 723 m, 741 s, 779 m, 810 s, 814 s, 817 s, 851 m, 967 s, 914 m, 974 m, 999 m, 1020 s, 1047 m, 1088 m, 1125 s, 1192 m, 1244 vs, 1273 vs, 1290 vs, 1360 s, 1396 s, 1429 m, 1449 m, 1485 s, 1530 m, 1570 s, 1582 s, 1618 m, 3038 w, 3076 w, 3291 m, 3308 m, 3362 m, 3389 m.

Anal. calcd. for $C_9H_6N_4O_3$ (218.0): C 49.55, H 2.77, N 25.68; Found: C 49.74, H 2.84, N 25.76.

2.1.9. 7-Nitro-*N*-((1-(3-(triethoxysilyl)propyl)-1*H*-1,2,3-triazol-4-yl)methyl)benzo[*c*][1,2,5]oxadiazole-4-amine (10)

7-Nitro-*N*-(prop-2-yn-1-yl)benzo[*c*][1,2,5]oxadiazole-4-amine (**6**, 250 mg, 1.15 mmol) and copper iodide (274 mg, 1.44 mmol) were dissolved in dry THF (10 mL). Then a mixture of diisopropylethylamine (742 mg, 5.75 mmol) and (3-azidopropyl)triethoxysilane (**7**, 356 mg, 1.44 mmol) was added and the mixture was stirred at room temperature for 16 h. Thereafter the reaction mixture was heated to 60 °C for 1.5 h. Thereafter, deionized water (80 mL) was added to the reaction mixture and the aqueous layer was extracted with dichloromethane (4 × 125 mL). The combined organic layers were dried with anhydrous magnesium sulfate and the solvent was removed in vacuo. The residue was purified by chromatography on silica gel (*n*-hexane/ethyl acetate 1:1) to give 7-nitro-*N*-((1-(3-(triethoxysilyl)propyl)-1*H*-1,2,3-triazol-4-yl)methyl)benzo[*c*][1,2,5]oxadiazole-4-amine (**10**, 233 mg, 0.500 mmol, 44%) as an orange solid.

Mp 95-98 °C.

R_f (*n*-hexane/ethyl acetate 1:5) = 0.47.

^{1}H NMR (300 MHz, $CDCl_3$): δ = 0.54-0.64 (m, 2 H), 1.20 (t, ^3J = 7.0 Hz, 9 H), 1.97-2.10 (m, 2 H), 3.80 (q, ^3J = 7.0 Hz, 6 H), 4.39 (t, ^3J = 7.2 Hz, 2 H), 4.79-4.87 (m, 2 H), 6.37 (d, ^3J = 8.6 Hz, 2 H), 7.02 (s, 1 H), 7.67 (s, 1 H), 8.47 (d, ^3J = 8.5, 1 H).

^{13}C NMR (75 MHz, $CDCl_3$): δ = 7.5 (CH_2), 18.3 (CH_3), 24.2 (CH_2), 39.5 (CH_2), 52.7 (CH_2), 58.6 (CH_2), 77.2 (C_{quat}), 99.6 (CH), 122.2 (CH), 124.7 (C_{quat}), 136.2 (CH), 143.2 (C_{quat}), 143.8 (C_{quat}), 144.3 (C_{quat}).

EI: *m*/*z* = 465.1 ([M]$^+$), 421.1 (10), 420.1 (37), 419.1 ([M]$^+$ - EtOH, 100).

IR (ATR): $\tilde{\nu}$ / cm^{-1} = 608 w, 654 m, 681 m, 723 m, 741 s, 779 s, 833 w, 858 m, 905 s, 955 s, 995 s, 1011 s, 1032 m, 1051 s, 1074 vs, 1099 vs, 1165 s, 1188 m, 1217 s, 1240 m, 1273 s, 1290 m, 1317 s, 1364 m, 1402 w, 1443 m, 1460 w, 1503 s, 1531 m, 1558 w, 1578 s, 1622 s, 2886 m, 2926 m, 2974 m, 3080 m, 3127 m, 3364 s.

Anal. calcd. for $C_{18}H_{27}N_7O_6Si$ (465.5): C 46.44, H 5.85, N 21.06; Found: C 46.70, H 5.85, N 20.77.

2.2. Synthesis of the dye-functionalized MCM-41 hybrid materials

2.2.1. Synthesis of Nile red-functionalized hybrid materials 8@MCM

Similar as described in [1] for the synthesis of the Nile red (NR) functionalized hybrid materials by postsynthetic grafting four different stock solutions of **8** in ethanol were prepared: c_1(**8**) = 49.0 μM, c_2(**8**) = 0.610 mM, c_3(**8**) = 5.00 mM, c_4(**8**) = 5.72 mM.

The solution of precursor **8**, commercially available MCM-41, and ethanol were subsequently added to the reaction vessel and stirred at room temperature for 20 h, followed by stirring at 80 °C for 24 h (for experimental details see Table S1). The obtained suspensions were centrifuged (10 min, 4000 rpm), decanted and resuspended in ethanol (20 mL) and 2 M aqueous hydrochloric acid solution (1 mL), upon which the red suspensions turned blue. After heating to 80 °C for 24 h the reaction mixtures were centrifuged, the solids transferred into a Soxhlet extraction thimble and extracted with hot ethanol over a period of 48 h. The obtained powders were washed with triethylamine (2 mL) and ethanol (20 mL), leading to a color change back to red. The solids were washed with ethanol (3 × 20 mL) and centrifuged each time as described until the supernatant reached pH 7. The obtained violet powders were dried at 60 °C and 10^{-3} mbar for 3 d to mass constancy.

Table S1: Experimental details of the synthesis of Nile red-grafted MCM-41 hybrid materials **8@MCM**.

Sample	Applied loading of hybrid with **8** [μmol·g^{-1}]	Determined loading of hybrid with **8** [μmol·g^{-1}]	Volume of stock solution [mL]	Mass of MCM-41 [g]	Volume of ethanol [mL]	Yield of NR grafted MCM-41 hybrid materials **8@MCM** [g]
**8@MCM-1**	1.0	0.6	8.91 of c_1(**8**)	0.437	6.10	0.408
**8@MCM-2**	2.5	1.5	0.50 of c_3(**8**)	1.00	14.5	0.560
**8@MCM-3**	5.0	2.9	14.3 of c_2(**8**)	1.75	0.70	1.64
**8@MCM-4**	10	5.9	2.00 of c_3(**8**)	1.00	13.0	0.624
**8@MCM-5**	15	8.8	3.00 of c_3(**8**)	1.00	12.0	0.752
**8@MCM-6**	20	12	3.50 of c_4(**8**)	1.00	11.5	0.802
**8@MCM-7**	20	12	14.3 of c_2(**8**)	0.437	0.70	0.396
**8@MCM-8**	30	18	5.24 of c_4(**8**)	1.00	9.76	0.804
**8@MCM-9**	40	23	6.99 of c_4(**8**)	1.00	8.01	0.892

2.2.2. Synthesis of perylene and benzofurazane-functionalized hybrid materials 9@MCM and 10@MCM

For the synthesis of the hybrid materials **9@MCM-1** and **10@MCM-1** stock solutions of each precursor molecule in THF with concentrations of c_1(**9**) = 0.639 mM and c_1(**10**) = 1.40 mM were prepared. The perylene stock solution c_1(**9**) (0.67 mL) was transferred to a suspension of MCM-41 (750 mg) in THF (9.23 mL). Likewise the benzofurazane stock solution c_1(**10**) (0.63 mL) was transferred to a suspension of MCM-41 (750 mg) in THF (9.37 mL).

For the preparation of the perylene-functionalized hybrid materials **9@MCM-2** to **6** the amounts of precursor **9** added to suspensions of MCM-41 (750 mg) in THF (5 mL) are given in Table S2. Likewise for the preparation of the benzofurazane-functionalized hybrid materials **10@MCM-2** to **7** the amounts of precursor **10** added to suspensions of MCM-41 (750 mg) in THF (5 mL) are given Table S3.

The reaction mixtures were stirred at room temperature for 20 h and then they were heated to 80 °C for 24 h. The obtained suspensions were centrifuged (10 min, 4000 rpm), decanted and resuspended in ethanol (20 mL). The suspensions were heated to 80 °C for 24 h after which the suspensions were centrifuged (10 min, 4000 rpm) and decanted. The obtained powders were transferred into a Soxhlet extraction thimble and extracted with hot ethanol over a period of 48 h. The obtained powders were dried at 60 °C and 10^{-3} mbar for 3 d to mass constancy.

Table S2: Experimental details of the synthesis of perylene-grafted MCM-41 hybrid materials **9@MCM**.

Sample	Applied loading of hybrid with **9** [μmol·g^{-1}]	Determined loading of hybrid with **9** [μmol·g^{-1}]	n(**9**) [μmol]	m(**9**) [mg]	Yield of perylene grafted MCM-41 hybrid materials **9@MCM** [mg]
**9@MCM-1**	1.0	0.8	0.75	0.426	626
**9@MCM-2**	2.5	2.0	1.88	1.07	612
**9@MCM-3**	5.0	4.0	3.75	2.13	643
**9@MCM-4**	7.5	6.0	5.63	3.20	634
**9@MCM-5**	10	8.0	7.50	4.26	426
**9@MCM-6**	25	20	18.8	10.7	630

Table S3: Experimental details of the synthesis of benzofurazane grafted MCM-41 hybrid materials **10@MCM**.

Sample	Applied loading of hybrid with **10** [μmol·g^{-1}]	Determined loading of hybrid with **10** [μmol·g^{-1}]	*n*(**10**) [μmol]	*m*(**10**) [mg]	Yield of benzofurazane grafted MCM-41 hybrid materials **10@MCM** [mg]
**10@MCM-1**	2.5	1.2	1.88	0.874	649
**10@MCM-2**	5.0	2.3	3.75	1.75	665
**10@MCM-3**	10	4.6	7.50	3.50	618
**10@MCM-4**	15	6.9	11.3	5.24	555
**10@MCM-5**	20	9.3	15.0	6.99	609
**10@MCM-6**	25	12	18.8	8.74	666
**10@MCM-7**	35	16	26.3	12.2	622

[1] Börgardts, M.; Verlinden, K.; Neidhardt, M.; Wöhrle, T.; Herbst, A.; Laschat, S.; Janiak, C.; Müller, T. J. J. *RSC Adv.* **2016**, *6*, 6209-6222. doi:10.1039/C5RA22736D

[2] Qiu, H.; Wang, C.; Xu, J.; Lai, G.; Shen, Y. *Monatsh. Chem.* **2008**, *139*, 1357-1362. doi:10.1007/s00706-008-0863-y

[3] Skorobogatyi, M. V.; Pchelintseva, A. A.; Petrunina, A. L.; Stepanova, I. A.; Andronova, V. L.; Gaegov, G. A.; Malakhov, A. D.; Korshun, V. A. *Tetrahedron* **2006**, *62*, 1279-1287. doi:10.1016/j.tet.2005.10.057

[4] Zhao, Y.; Pirrung, M. C.; Liao, J. *Mol. BioSyst.* **2012**, *8*, 879-887. doi:10.1039/C2MB05471J
